# Interaction driven quantum Hall effect in artificially stacked graphene bilayers

**DOI:** 10.1038/srep24815

**Published:** 2016-04-21

**Authors:** Muhammad Zahir Iqbal, Muhammad Waqas Iqbal, Salma Siddique, Muhammad Farooq Khan, Shahid Mahmood Ramay, Jungtae Nam, Keun Soo Kim, Jonghwa Eom

**Affiliations:** 1 Faculty of Engineering Sciences, GIK Institute of Engineering Sciences and Technology, Topi 23640, Khyber Pakhtunkhwa, Pakistan; 2 Department of Physics & Graphene Research Institute, Sejong University, Seoul 143-747, Korea; 3 Department of Physics & Astronomy, Georgia State University, Atlanta, GA 30303, USA; 4 Department of Physics, College of Science, Majmaah University, Al-Zulfi 11932, Saudi Arabia; 5 Department of Bioscience & Biotechnology, Sejong University, Seoul 143-747, Korea; 6 Physics & Astronomy Department, College of Science, King Saud University, Riyadh 11451, Saudi Arabia

## Abstract

The honeycomb lattice structure of graphene gives rise to its exceptional electronic properties of linear dispersion relation and its chiral nature of charge carriers. The exceptional electronic properties of graphene stem from linear dispersion relation and chiral nature of charge carries, originating from its honeycomb lattice structure. Here, we address the quantum Hall effect in artificially stacked graphene bilayers and single layer graphene grown by chemical vapor deposition. The quantum Hall plateaus started to appear more than 3 T and became clearer at higher magnetic fields up to 9 T. Shubnikov-de Hass oscillations were manifestly observed in graphene bilayers texture. These unusual plateaus may have been due to the layers interaction in artificially stacked graphene bilayers. Our study initiates the understanding of interactions between artificially stacked graphene layers.

The robust electronic properties of graphene stem from linear dispersion relation, containing massless Dirac fermions and chiral nature of charge carriers. This behavior of graphene originats from its honeycomb lattice structure. Bernal stacked bilayers graphene consists of massive Dirac fermions spectrum, which is demonstrated by two pairs of parabolic bands^1^,^2^,^3^. The massless Dirac spectrum could be expectedly present in bilayers graphene if both layers were precisely placed with AA-stacking symmetry^4^,^5^. The staking of more graphene layers are useful for vertical transport which enhance the spin signal^6^,^7^. The structural distortion in the layers is manifested to the formation of correlated states and therefore the distortion can be created by applying strain^8^,^9^, as stacking one layer on top of another layer^10^,^11^,^12^,^13^,^14^,^15^. This artificial stacking of graphene bilayers is usually expected to be unstable towards symmetry breaking due to the twist angle. The recent reports showed that a very small distribution is sufficient enough to generate a completely new electronic spectrum with broken symmetry^16^. The interlayer Coulomb interactions and tunneling effects of the two closely spaced graphene layers may lead to a new interesting phenomena. The new phenomenon is similar to the bilayers of two dimensional electron gas; which not present in individual layers^17^,^18^,^19^. Perhaps due to the Fermi surface of carbon it is possible where the honeycomb lattice in graphene are centered at nonzero K vectors^20^ and relative disparity between the layers resulting in weak coupling^21^. The effect of symmetry breaking of graphene and its significant in electronic transport properties is an enduring topic to identify the various ground states.

The electrical transport such as quantum Hall effect in single and Bernal stacked bilayers graphene has been explored to a large extent. However, it is interesting to investigate the electronic transport properties of artificially stacked graphene layers. Here we report the electronic transport properties of artificially stacked chemical vapor deposition grown graphene bilayers. We have observed that the quantum Hall effect consisting of various plateaus with non-integer quantized values at 4.2 K, however a typical massless Dirac fermions spectrum has observed in a single layer graphene. The clear quantum Hall plateaus started to appear from 3 T in graphene bilayers and became more prominent at higher magnetic fields. The Shubnikov-de Hass (SdH) oscillations were observed as well in graphene bilayers texture.

## Results and Discussion

**Figure 1:**
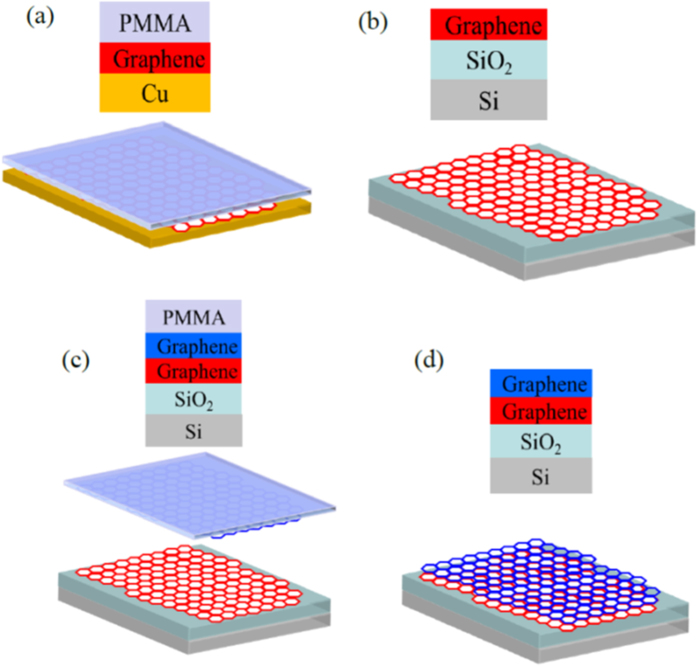
Schematic of artificially stacked graphene bilayers with top layer having twist angle. (**a**) Graphene on Cu file with PMMA coating. (**b**) Graphene transferred on the SiO_2_/Si substrate. (**c**) Second graphene layer coated with PMMA subsequently transferred to the first layer. (**d**) Graphene bilayers transferred to the SiO_2_/Si substrate.

Figure 1 shows a schematic steps and the optical image of two mis-oriented graphene layers on SiO_2_/Si substrate. Figure 1(a) shows the CVD grown graphene on Cu file with PMMA coating. The second step is the transferred of graphene to SiO_2_/Si substrate as displayed in Fig. 1(b). The second graphene layer coated with PMMA is subsequently transferred to the first graphene layer to make it bilayers shown in Fig. 1(c). The complete schematic of graphene bilayers after removing PMMA is presented in Fig. 1(d). The bottom layer of graphene has shown in red color honey combs while the top graphene layer has represented by the color blue.

**Figure 2:**
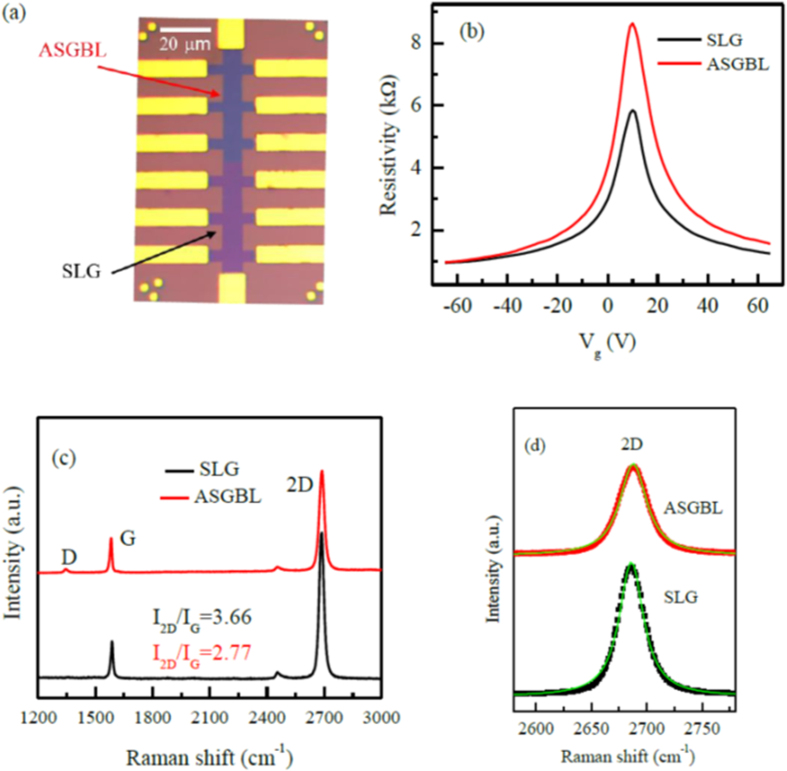
(**a**) Optical microscope image of graphene Hall bar patters of artificially stacked graphene bilayers (ASGBL) and single layer graphene (SLG) region. (**b**) Resistivity as a function of back gate voltage (V_g_) for ASGBL and SLG. (**c**) Raman spectra of ASGBL and SLG region shows the 2D-to-G ratio of 3.66 and 2.77 cm^−1^, respectively. (**d**) Lorentz fitting to the 2D peaks of ASGBL and SLG to estimate the full width half maxima.

The optical micrograph image of complete Hall bar device structure with Au electrodes has shown in Fig. 2(a). Where the dark part of Hall bar device represents the bilayers graphene region and slightly the light color indicate the single layer graphene. The resistivity as the function of back gate voltage for artificially stacked graphene bilayers (ASGBL) device and a single layer graphene (SLG) region has shown in Fig. 2(b). The field effect hole and electron mobility values of the single layer region of graphene device have found to be around 2363 and 2060 cm^2^/Vs, respectively; while the field effect hole and electron mobility values of the double-layer region of graphene device at 0 T appeared to be about 2058 and 1625 cm^2^/Vs at temperature of 4.2 K, respectively. Figure 2(c) shows the Raman spectra of SLG and ASGBL. The 2D/G peak intensity ratio (I_2D_/I_G_) value is ~3.66 for the case of SLG and ~2.77 for ASGBL as shown in the Fig. 2(c). The D-like peak is observed due to the twist angle between two graphene layers and similar kind of peaks have been observed previously in the twisted bilayers graphene as reported by C. C. Lu *et al.*^22^. The full width half maxima (FWHM) of 2D peaks for both SLG and ASGBL estimated by Lorentz fitting are about ~26 and ~29 cm^−1^, respectively as shown in Fig. 2(d).

**Figure 3:**
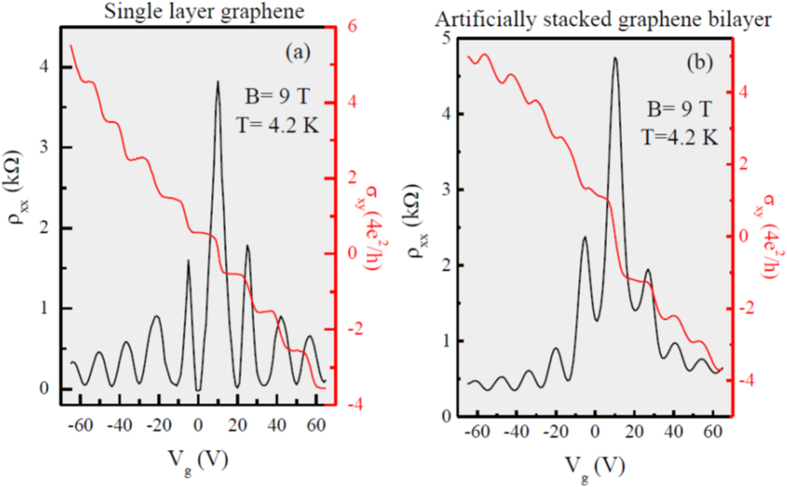
The longitudinal resistivity and Hall conductivity of as a function of applied back-gate voltage (V_g_) with magnetic field of 9 T at 4.2 K (**a**) Single layer graphene (**b**) Artificially stacked graphene bilayers.

Figure 3(a) shows the longitudinal resistivity (ρ_xx_) and Hall conductivity (σ_xy_) of SLG as a function of applied back-gate voltage (V_g_), with magnetic field (B) of 9 T at 4.2 K. The result showed a strong evidence of quantum Hall effect (QHE) with conductivity plateaus appeared at ±2, ±6, ±10, ±14, +18, together with resistivity minima, consistent with the Landau level spectrum expected for SLG graphene^23^. In the case of ASGBL has also showed the QHE plateaus with different conductivity values as shown in Fig. 3(b). These plateaus revealed an unusual trend, which is different from the single layer or the Bernal stacked bilayers graphene^2^,^23^. Therefore, these observations refer to a perturbed system that may be due to interaction between two graphene layers.

**Figure 4:**
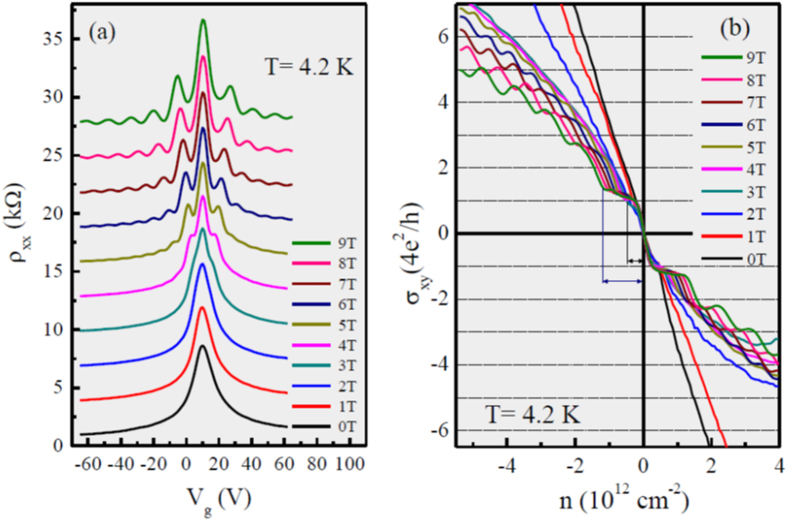
(**a**) The longitudinal resistivity as a function of the back-gate voltage and (**b**) Hall conductivity as a function of the charge carrier density from 0 to 9 T (with step of 1 T) at 4.2 K.

Figure 4(a) shows the longitudinal resistivity as a function of applied back-gate voltage from 0 to 9 T at 4.2 K. At the lower magnetic field up to 3 T no evident peak is observed, the resistivity plots are similar as 0 T. However, under application of high magnetic fields, transport measurements show a spectrum of symmetric oscillations on the each side of Dirac point with resistivity minima by increasing the applied back-gate voltage. At fields larger than 3 T, the plateaus are started to appear and became clearer at higher magnetic fields up to 9 T, indicative of the high quality of our sample. Figure 4(b) shows the Hall conductivity as a function of the charge carrier density induced by the applied back-gate voltage in the magnetic field range from 0 to 9 T (with step of 1 T), at 4.2 K. The total density is calculated from n = C_g_(V_g_ − V_Dirac_)/e, where C_g_/e = 7.19 × 10^10^ cm^−2^V^−1^ and V_Dirac_ = +10 V is the offset voltage to reach charge neutrality^24^. The transverse transport measurements show the development of quantum Hall states (QHSs) which are consistent with vanishing resistivities as shown in Fig. 4(a).

**Figure 5:**
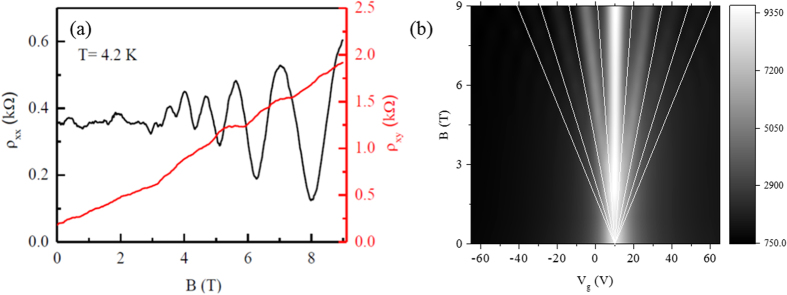
(**a**) The quantum Hall measurements of the longitudinal (ρ_xx_) and the Hall resistivity (ρ_xy_) as a function of magnetic field at local back gate voltage of −40 V. (**b**) The contour plot of longitudinal resistivity (ρ_xx_) as a function of V_g_ and B.

Figure 5 (a) shows a comparison of quantum Hall measurements of the longitudinal (ρ_xx_) and the Hall resistivity (ρ_xy_) as a function of magnetic field at local back gate voltage of −40 V. Interestingly, this complex system of artificially stacked graphene bilayers shows the Shubnikov-de Haas oscillation. The resulting data (ρ_xx_ vs. B) reveal SdH oscillations which starts from magnetic fields as low as B ~3 T and it is consistent with Fig. 3 data, where QHSs are visible at the same B fields. Furthermore, the longitudinal resistivity against V_g_ and B data which follows the quantum Hall states randomly as a monolayer (ν = ±2, 6, 10, 14, 18, 22, …) or bilayers (ν = ±4, 8, 12, 16, 20, 24, …). The contour plot of longitudinal resistivity (ρ_xx_) as a function of V_g_ and B is shown in Fig. 5(b). The black lines are representing observed filling factor positions of ASGBL at (ν = ±4, 6, 10, 14, …). Some previous studies of artificially stacked or twisted bilayers graphene describe the parallel conduction though each monolayers, while the other results follow the four-fold or eight-fold degeneracy^25^,^26^,^27^. As the unusual perturbation due to the layer interaction has being predicted by the angle resolved photoelectron spectroscopy (ARPES). Experiments that refer to the symmetry-broken bilayers graphene as reported by K. S. Kim *et al.*^16^ and theoretically predicted by M.-Y. Choi *et al.*^28^. Although, the theoretical observation of landau-level spectra in twisted bilayers graphene has reported by Z. F. Wang *et al.*^29^. In the light of above mentioned experiments the hierarchy of Dirac fermions are significantly related to charge transport due to the combination of massive and massless fermions and the quantum transport phenomenon such as valley Hall effect in multiband complex system.

## Conclusion

In summary, we have studied the structural and electrical transport properties of chemical vapor deposition grown single layer graphene and artificially stacked graphene bilayers. The typical massless Dirac fermions spectrum is observed in the quantum transport of single layer graphene, however artificially stacked graphene bilayers texture follows the quantum Hall states randomly as a monolayer or bilayers at 4.2 K. The hierarchy of Dirac fermions are significantly related to charge transport due to the combination of massive and massless fermions. The unusual Dirac fermion spectrum may have possibly caused by the layers interaction in artificially stacked graphene bilayers.

## Methods

### Graphene growth and device fabrication

Graphene film was grown on 25-μm-thick copper foils from Alfa Aesar (99.8% pure) via thermal CVD. A mechanically polished and electropolished copper foil was inserted into the CVD furnace. The furnace was evacuated to ~10^–4^ Torr, and the temperature rose to 1010 °C with H_2_ gas flow (~10^–2^ Torr). After the temperature stabilized at 1010 °C, CH_4_ and H_2_ (20 and 5 standard cubic centimeters per minute, respectively) were injected into the furnace to synthesize the graphene for 8 min, after which the sample was cooled at a rate of 50 °C/min to room temperature^30^. The graphene film grown on Cu foil was transferred to a Si substrate by the wet transfer method. The Cu foil was spin-coated (850 rpm for 10 s, 2500 rpm for 30 s) with a thin layer of polymethylmethacrylate (PMMA). Then, the bottom Cu foil was removed by etching in a 1 M solution of ammonium persulfate (APS, (NH_4_)_2_S_2_O_8_), and the PMMA membrane was washed with deionized water. Next, the graphene film with the PMMA membrane was transferred to the p-doped Si substrate having a top 300 nm-thick layer of SiO_2_. The graphene layers transferred onto the Si/SiO_2_ substrate were heated at 80 °C for 10 min to dry and then put in acetone for one day to completely dissolve the PMMA layer^30^,^31^. An artificial double-layer graphene was formed by subsequent transfer of another layer onto the first layer of graphene. After transferring and making the Hall bar of one graphene, then half part of it was removed with a combination of electron-beam lithography and oxygen plasma treatment, i.e. electron-beam lithography was used to expose the specified area of the graphene that was intendant to be removed and oxygen plasma treatment was used to engrave that specified area. Each step of the process was carefully examined by microscope to verify the complete etching of graphene in the desired area. The second graphene layer was transferred thereafter and made a Hall bar with careful adjustments it was then aligned exactly on to the engraved graphene Hall bar. Therefore, we were able to examine the characteristics of double and single-layer graphene. The Cr/Au (5/30 nm) contacts were coated by using a thermal evaporation system.

### Device characterization and measurement setup

Raman spectra were measured with a Renishaw microspectrometer over a wavenumber range from 1100 to 3200 cm^–1^, with a laser wavelength of 514.5 nm. The spot size was 1 μm and the power was kept at 1.0 mW to avoid local heating. The electrical and magneto-transport properties of graphene junctions were measured using ac lock-in techniques at frequency of 11.7 Hz with the root-mean-square current amplitude of 50 μA. Low temperature measurements were carried out in liquid helium cryostat (down to 4.2 K) with magnetic field up to 9 T. Lake Shore 331 temperature controller was utilized to modulate and control the temperature range.

## Additional Information

**How to cite this article**: Iqbal, M. Z. *et al.* Interaction driven quantum Hall effect in artificially stacked graphene bilayers. *Sci. Rep.* **6**, 24815; doi: 10.1038/srep24815 (2016).
